# Aging Parents and the Ties That Bind: Intergenerational Relationship Quality Among Culturally Diverse Canadian Families

**DOI:** 10.1177/00914150241240120

**Published:** 2024-03-20

**Authors:** Barbara A. Mitchell, Samantha Teichman

**Affiliations:** 1 Department of Sociology/Anthropology, Simon Fraser University, Burnaby, BC, Canada; 2 Department of Gerontology, Simon Fraser University, Vancouver, BC, Canada

**Keywords:** aging families, relationship quality, cultural diversity, life course theory, health & well-being

## Abstract

Drawing from a life course perspective, this paper examines mid/later-life parent–child relationship quality among ethnically diverse families. Focus is on the role of culture, child, and parental characteristics. Data are drawn from a study of 588 parents aged 50+ of a least one child aged 19–35 who reside in Metro Vancouver, B.C. from four Canadian cultural groups: British, Chinese, Persian/Iranian, and South Asian. Using OLS regression methods, we use two dependent variable scales: positive and negative support/interaction appraisals of the relationship. The positive relationship quality scale is associated with South Asian versus British-Canadian parents, mothers, those with greater income satisfaction, and daughters. The negative scale is inversely associated among South Asian versus British-Canadian parents, income satisfaction, parental health, and being unpartnered (vs. partnered). Interaction effects are found between gender and ethnicity. Implications for theorizing and applied recommendations for those who work with culturally diverse aging families are discussed.

Parenting represents one of the most meaningful and significant adult life experiences (Cohen, 2015). Parent–child bonds and the quality of these relationships are also fundamental to individual and family psycho-social health and well-being over the life course (Reczek & Zhang, 2016; Thomas et al., 2017). These ‘ties that bind’ also typically demonstrate both positive and negative aspects or qualities and can be both a source of comfort and support and/or a source of conflict or irritation (Pitzer et al., 2011). Moreover, parental experiences have changed in contemporary society due to shifting socio-demographic and immigration patterns. Transformations in the transition to adult and parental roles also contribute to more culturally diverse family experiences in North American society. In particular, we are undergoing a longevity revolution because of rapid population aging in tandem with rising immigration to Canada from many non-Westernized countries (Statistics Canada, 2022). In short, these emergent family trends can potentially and dramatically shape the quality and nature of aging parents’ relations with their adult children.

Yet to date, while a multitude of studies have been conducted on parent–child relationships in younger families and some have included some racialized or minority groups, especially in the U.S. (e.g., Hamel et al., 2022; Yahirun & Kroeger, 2019; Zhang & Grant, 2023), virtually no known Canadian studies have been devoted to ties that extend into later life and that are culturally diverse. Indeed, only scant Canadian attention has been paid to parent–child relationships in older-aged Chinese, South Asian, and Italian families, and this focus has been on such topics of happiness in parental roles and intergenerational conflict and its sources (e.g., see Mitchell, 2010). This information is especially timely and important given our rapidly aging population and since almost one in four Canadian people (23%) are, or have been, landed immigrants or permanent residents. Most of these recent immigrants have also originated from Asia, including the Middle East, representing collectivist cultures (Statistics Canada, 2022).

This dearth of information on intergenerational relationship quality in culturally diverse Canadian aging families is problematic for several reasons. First, there has been a rapidly rising immigrant population originating from countries such as Iran (formerly known as Persia). Relatively few Iranians lived in Canada before the 1979 Iranian Revolution. A large stream of migrants arrived in the years right after this event and an even larger stream more recently (Rahnema, 2020). These Persian/Iranian residents are diverse (similar to other highly populated Asian-Canadian communities such as the Chinese and South Asian), although they may share some commonalities in their cultural, socio-political, and religious heritage (Rahnema, 2020). This diaspora has faced considerable personal challenges upon resettlement, including racism and discrimination, similar to other non-White groups. These experiences have been found to negatively affect intimate and family relationships and create some additional stress and strain. Thus, in a rapidly aging and multicultural society, knowledge of ethnocultural variations in mid/later-life Canadian parent–child relations is timely. This knowledge can also provide some valuable information to academics, educators, healthcare professionals, (e.g., family counselors) and community organizations working with culturally diverse aging families.

In addition, many broader familial and socio-economic shifts have occurred in family life that can differentially shape parent–child relationships. Family transitions, such as young adult children leaving home and forming their own families, are increasingly extended or delayed in many industrialized countries. Coresidence can build bridges across the generations (e.g., through prolonged support), or alternatively, negatively impact these relationships (Alder & Lenz, 2023). Indeed, many young adult children (regardless of social class and ethnicity) continue to be dependent (socially, financially) upon their parents, and many are living at home until later ages (Kornrich & Furstenberg, 2013; Maroto, 2017). This extended parenting can potentially create intergenerational and dyadic problems since it typically occurs when parents are in the process of trying to plan or transition to retirement (Boveda & Metz, 2016; Klassen, 2013). Yet, it is commonly known that some traditional ethnic groups, such as South Asians, prefer or favor extended or multigenerational households due to economic reasons and cultural traditions. Additional factors influencing intergenerational relationships between aging parents and adult children are also found to include aspects such as gender, age, socio-economic status, and health status (Torabian et al., 2022).

In light of these changing trends in aging family life and gaps in knowledge, the present study is designed to explore the parent–child relationship quality of Canadian parents aged 50+ with at least one child (aged 19–35) in terms of ethnocultural family background (ethnic group identity; years living in Canada, religiosity); young adult characteristics (gender, age, marital status, main activity, living arrangement); and parental socio-demographic/health-related variables (e.g., gender, age, marital status, income satisfaction, health status). Theoretical implications and applied recommendations for those working with culturally diverse aging families (e.g., health and social service professionals) and community providers are also discussed.

## Theoretical Perspective

A socio-cultural life course theoretical lens guides and frames this study (Elder, 1998, 2000; Giele & Elder, 1998; Mitchell, 2003; Zittoun, 2012). This perspective represents a highly popular multidisciplinary approach dominant in social gerontology, with strong roots in the sociology of aging and age stratification. This framework has also evolved to increasingly incorporate many insights into how culturally diverse families experience their lives as they develop, mature, and change over time (Zittoun, 2012). The life course of aging families is viewed as embedded in, and shaped by, their relative historical, socio-cultural, and geographic location, as well as other family contextual factors, such as socio-demographic characteristics and family resources (Hendricks, 2012). Hence, the diverse cultural context(s) and geographical locale in which contemporary later-life parenting occurs can shape/re-shape families’ behaviors, values, and practices. For example, cultural factors can influence ideas about what constitutes ‘happy’, ‘successful’, or ‘ideal’ parent–child relationships as families grow older. Thus, families can be conceived as dynamic micro-social groups with some shared history who interact within an ever-changing macro-social and cultural environment (Bengston & Allen, 1993).

As noted by Sechrist et al. (2012), over the past century, two dominant themes emerged in the study of parent–adult child relations. One perspective emphasizes family solidarity and views adult children and their parents as a significant source of support (emotional and instrumental) for one another. Conversely, another lens highlights the potential for intergenerational conflict, including the abandonment of aging parents. This latter viewpoint is assumed to reflect generalized societal fears about the health and well-being of older adults, although more recent theorizing emphasizes the need to incorporate both positive and negative aspects by drawing upon the concept of intergenerational ambivalence (Lüescher & Pillemer, 1998). Yet, while many studies have accumulated to demonstrate that ambivalence is very prevalent in later-life families, the application of evolving conceptual frameworks that incorporate both negative and positive aspects to non-White ethnically diverse aging families has been sparse. For instance, in traditional ethnic groups (e.g., Asian, Middle-Eastern), there may be strong norms of filial piety, respect, and honor, which in turn, can buffer stressful experiences of social life.

A core tenet of the life course perspective emphasizes how aging family development and human lives are intimately intertwined and reciprocally connected in society (Elder, 1998). Although ‘culture’ is not a monolithic entity and it can be multidimensional, it is well known that ethnic and cultural contexts can affect the intergenerational experiences of aging families. Indeed, ethnic culture is “a collective cognitive scheme with the power to structure social and family relationships” (Silverstein et al., 2012, p. 300). As a construct, ethnic culture can shape aspects such as family values, identity, race, religious orientation, and immigrant status (Silverstein et al., 2012). It can also shape norms and expectations regarding the ‘appropriate’ nature and timing of life course events, including social timetables, transitions to new roles, and significant life events (e.g., children's home leaving, migration to a new country). In a multicultural society such as Canada, these ethnocultural contexts can therefore differentially impact individual experiences and influence parent–child relationships in positive or negative ways. For example, among recent immigrant families, a relatively short period of settlement can potentially create stress and intergenerational conflict and negatively affect the quality of the parent–child relationship.

Moreover, other socio-demographic characteristics and life course contextual environments tied to both the adult child (e.g., their gender, age, marital status, main activity, living arrangement) and the aging parent (e.g., gender, age, income, health, marital status) can play a role in shaping the quality of later-life intergenerational relations. For example, the gendered nature of kin-keeping plays a key role for mothers in establishing the strength and quality of intergenerational ties relative to fathers (Kalmijin et al., 2019). Also, from a life course perspective, relationship quality between adult children and aging parents becomes more complex as a result of many changes, including emerging health issues, evolving support exchanges, shifting living arrangements, and marital status changes (Ahmad & Reid, 2008; Carr et al., 2014; Kamali et al., 2020; Lee et al., 2021; Lewin, 2017; Umberson & Williams, 2005).

The following section reviews the empirical literature on our focused research question. Given our special interest in ethnocultural diversity, we begin with a review of studies dealing with ethnicity, religiosity, and immigration factors. This will be followed by a synthesis of research studies on child characteristics and other parental variables and their associations with relationship quality, with emphasis on those factors that are included in our data set and analysis.

### Cultural Variables: Ethnicity, Religiosity, and Immigration Factors

It is well-documented that ethnic group identity plays a vital role in shaping family relationships and development over the life course (Albertini, Mantovani et al., 2019; de Figueiredo, 2014; Fingerman et al., 2020; Shariff, 2009). Individualism and collectivism are core values that distinctly set apart Eastern and Western cultures. Eastern cultures typically emphasize collectivism and filial piety, which are assumed to create strong interdependent family ties across the generations. In contrast, Western cultures tend to prioritize individualism and independence, leading to more emphasis on personal autonomy and reliance on formal support systems for aging family members (Bedford & Yeh, 2019; Bengtson, 2000; Connidis & Barnett, 2018; Sadeghi et al., 2012; Shariff, 2009; Wister & McPherson, 2019). Thus, we might expect that intergenerational relationship quality will be more positive/less negative among parents originating from cultures (i.e., Chinese, Persian/Iranian/South Asian) that accentuate familism and supportive parent–child bonds over personal needs and goals.

Religion's impact on family relationships also tends to vary among cultural groups, influencing values, lifestyles, and expectations. In less religious families, young adults generally have more personal freedom and less religious authority influence (Fingerman et al., 2020; Kalmijn, 2019). In more collectivist cultures, such as the Chinese culture, traditional beliefs such as Confucianism contribute to strong family values and religious influence (Bedford & Yeh, 2019; Ho, 1986). Similarly, South Asian culture, encompassing religions such as Hinduism, Islam, and Sikhism, underscores collective family dynamics through the emphasis on religious practices, rituals, and cultural identity (Ahmad & Reid, 2008; Shariff, 2009; Trieu, 2016; Zaidi et al., 2014). Persian/Iranian culture's predominant Islamic influence echoes these collective family values and practices, with long-established religious and cultural traditions (Karimi & Hiitola, 2019; Sadeghi et al., 2012). Strong religious beliefs can significantly influence family dynamics by fostering cohesion and impacting choices. Thus, although we are not examining religious differences by specific ethnic groups, we expect that generally, parents with stronger religious beliefs may perceive and report more favorable relationships with their children compared to those with weaker religiosity.

Additionally, although this study does not focus on immigrant families per se, and not all of our sample includes immigrants (especially the British group), in general, the number of years living in Canada can have a significant impact on parent–adult child relationships; affecting communication patterns, intergenerational dynamics, cultural identity, and family roles (Baykara-Krumme & Fokkema, 2019; Fingerman et al., 2020; Karimi & Hiitola, 2019). On the one hand, we might expect that parents from individualistic cultures (e.g., British-Canadian) will have less positive relations than those from more collectivist groups, including many immigrant groups. Yet, over time, immigrant parents and adult children may develop different language proficiency levels, impacting the ease and depth of communication, although this could potentially lead to misunderstandings or reduced emotional closeness (Cox et al., 2021; Nesteruk et al., 2015). Yet, other research suggests that more recent resettling to North America can lead to conflicts between generations due to varying cultural perspectives and expectations (e.g., see Choi et al., 2008; Phinney et al., 2000).

We might predict, therefore, that those living in Canada for shorter periods—relative to longer established families—will generally perceive less positive relationships with their adult children. More newly arrived parents may strongly uphold their traditional culture, which can create intergenerational conflict and reduce emotional closeness. Thus, we hypothesize that generally, parents who have lived in Canada for fewer years will be more likely to report less positive/more negative relationship quality than those who have lived in Canada for more years.

### Child Characteristics

The gender of the adult child can influence parent–child relationships in various ways. Research suggests that parent–daughter relationships often have perceived higher emotional closeness and communication frequency in comparison to parent–son relationships (Alford, 2021; Cao et al., 2019; Golish, 2000; Rastogi & Wampler, 1999; Shapiro, 2004). This could be a result of the type of support and assistance they provide, given that daughters (as well as daughter-in-laws in traditional cultures) tend to provide more caregiving support to parents in later life while sons might be more focused on instrumental support and financial assistance (Fingerman et al., 2020; Torabian et al., 2022; Vergauwen & Mortelmans, 2021).

Relatedly, the age and living arrangement of the adult child can shape the parent–adult child dyad. For instance, younger-aged adult children and those who live at home may rely more on their parents for guidance and day-to-day support (Fingerman, 2017; South & Lei, 2015; Stone et al., 2013), which can have both positive and negative implications for the parent–adult child relationship. Indeed, the effects of co-residence on parental well-being are intricate, offering support but causing strains (Fingerman et al., 2020; Riley & Bowen, 2005). Yet, it is important to consider that coresidence patterns, differ in Western and non-Western cultures. For example, in the West, adult children leaving home signifies maturity, family “success” and norms of independence (Mitchell & Lovegreen, 2009). However, generally, we expect that intergenerational relationship quality will be lower for parents with both younger-aged children and for those that co-reside in the same household due to issues of dependency that can create more daily stress and strain.

Additionally, as adult children age and form their own families, they might seek more independence (Furstenberg, 2010), which can affect the perceived closeness between them and their aging parents. Relatedly, the marital status of the adult child can affect the quality of relationships. Married adult children might prioritize their immediate family, potentially leading to reduced frequency of interactions and support for their parents (Clarke et al., 1999; Connidis & Barnett, 2018; Furstenberg, 2010). Conversely, unmarried adult children could maintain stronger connections with their parents and be more available for caregiving and various kinds of support (Sarkisian & Gerstel, 2008). Also, the young adult's main activity, such as being a student, working professional, or being unemployed, is found to impact the time and energy that they have to invest in their family relationships (Albertini, Gasperoni et al., 2019). Thus, we might expect that young adults who are engaged in main activities that denote less parental dependency (e.g., are employed), will be perceived more favorably by parents.

### Parent Characteristics

The gender of the parent can shape parent–adult child relationships. Mothers typically exhibit greater positivity, support, and affection towards their children compared to fathers, resulting in children expressing stronger emotional attachment and closeness to their mothers than to their fathers (Alford, 2021; Pillemer et al., 2012; Suitor et al., 2006; Umberson, 1992; Ward, 2008). This can be explained by mothers’ heightened commitment to the parent–child connection, driven by their stronger investment in the relationship and societal norms that associate femininity with the role of motherhood (Pillemer et al., 2012). Therefore, we hypothesize that mothers will report more positive relationships with their adult children than fathers.

Growing parental age alters social support availability, influenced by shifts in work ties post-retirement, health-related challenges, and family structure changes (Carr et al., 2014). Older parents might require more support and caregiving from their adult children, leading to a reversal of roles (Connidis & Barnett, 2018; Fingerman et al., 2020; Riley & Bowen, 2005). This change in roles can cause strain within the relationship as aging parents grapple with a loss of control and adult children take on more caregiving responsibilities while juggling their personal lives (Conway, 2019; Morgan & Brazda, 2013). This observation highlights how older age intersects with declining health and other status changes, impacting the parent–adult child dyad. Thus, we expect that parents reporting poorer health, as well as older-aged parents will report more positive relationships with their adult children than healthier and younger-aged parents.

Changes in marital roles and status for the aging parents can have more positive effects. Although research suggests that some martial changes earlier in life (i.e., divorce) can weaken parent–child bonds (e.g., see Kalmijin et al., 2019), in later life, divorced or widowed parents may increasingly lean more on their adult children for companionship and support, potentially deepening relationships. Widowhood also leads to less ambivalence between parents and adult children (Fingerman et al., 2020). It tends to increase parents’ reliance on children while decreasing children's dependence (Ha et al., 2006). Therefore, we anticipate that unpartnered parents may perceive more positive relationships with their child(ren).

Financial difficulties are also consistently found to undermine the quality of parent–child relationships. In particular, dissatisfaction with one's income may reflect money problems, family stress, and strain, including issues related to flows of financial support to/from adult children (Mitchell et al., 2019). Thus, we predict that lower-income satisfaction will be associated with negative parent–adult child relationship quality.

### Interaction Effects

Gender and ethnicity can interact in complex ways to shape parent–adult child relationships. Cultural norms and values associated with ethnicity can influence the values and expectations placed on individuals based on their gender. For instance, in some cultures, gender roles may be more traditional, affecting how daughters and sons are socialized and the roles they are expected to fulfill within the family (Usita & Du Bois, 2005; Zaidi et al., 2014). Additionally, the intersection of gender and ethnicity can influence how parents pass down cultural traditions, norms, and values to their children, which may impact relationship closeness and support and role demands between parents and adult children. Thus, we include this interaction effect to further explore these associations.

## Methods

### Sampling and Data

This study utilizes data (*n* = 588) collected from the first author's Social Science and Humanities Research Council of Canada funded project entitled, “The Nexus of Intergenerational Ties in Young Adulthood and Parental Transitions to Adulthood”*.* This mixed-methods study was conducted in the Metro Vancouver area of British Columbia, Canada, between 2015 and 2017. The overarching objective of this project was to examine various family transitions across the life span (e.g., transitions to the empty nest and retirement), in addition to focusing on diversity in family relationships. We also were able to ask some more detailed questions on the parent–child relationship from the perspective of one parent, but only with one focal child (FC) if multiple children (due to budgetary and time restrictions).

To participate in the study, we had several study criteria. Specifically, participants had to be aged 50 or older; have at least one adult child aged 19–35; and primarily self-identify with one of four ethnocultural groups: British (English, Scottish, Welsh, Irish), Chinese, Persian/Iranian, or South Asian-Canadian (including Indo-East Indian Pakistan, Sri Lanka, Bangladesh, Afghanistan, Nepal, Bhutan, Maldives). These cultural groups were chosen because they represent large and/or rapidly rising visible minority groups living in predominantly Canadian urban centers.

Survey data were collected between March 2015 and November 2016. We used two recruitment methods: 1) “cold calls”—via random (using random number charts) selection of numbers based on local telephone directories (47.4%, *n* = 279); and 2) referrals/snow-ball sampling, posters, visits to local churches or community organizations (e.g., community centers, immigrant service societies), coffee shops, or shopping malls (52.6%, *n* = 309). This data collection approach was needed because there were many recruitment-related challenges during this time period (e.g., many people were transitioning to cell phone technology, concerns about telephone fraud, and generally low public trust in research). We also learned that many new immigrants to Vancouver (i.e., Persian/Iranian, South Asian, and Chinese groups) were reluctant to participate in research initiated through a “cold call” recruitment strategy due to language and cultural barriers (e.g., fears about possible political persecution). The average length of the interviews was 30–40 min, and we covered a range of family, social, psychological, economic, and demographic areas. Interviews were conducted in the participant's preferred choice of language by a trained interviewer who was fluent in English as well as that language.

Participants were also classified according to one of three living arrangements: (a) full nest (all children currently reside in the parental home); (b) emptying nest (at least one child has left home for four or more months and is currently not living at home; (c) empty nest (all children have left home for at least four months and none currently live at home). The interviewers also administered pre-retirement/retirement modules, depending on their stage of retirement (not retired, partially retired, completely retired). Thus, although a baseline questionnaire was used, we needed to tailor the wording for relevant items related to the different living arrangements and retirement stages (i.e., using past, present, or future tense). In addition, if the parent had more than one child eligible to serve as a focal child (FC), the interview randomly selected a “study child” (FC).

While we aimed to have similar quotas for each of the four ethnic groups, our sample included more South Asians (*n* = 194, 33%), followed by Persian/Iranians (*n* = 144, 25%), and equal numbers of British- and Chinese Canadian parents (*n* = 250; at 21% each). The ethnic origin of the ethnic clusters also indicated that the majority of British respondents were born in Canada; the Persian/Iranian respondents were born in Iran; most of the “Chinese” sample were born in either Mainland China or Hong Kong; and the majority of the South Asian sample emigrated from India. As shown in Table 1, the average parent age was approximately 60 (standard deviation = 6.6) and the average age of the focal child was 28 (standard deviation = 4.8). Most young adult children were non-partnered (64%) and employed (60%). In terms of gender, slightly over half of the parents were female (57%), although focal children were slightly more likely to be sons (53%). Finally, the living arrangements with children included: 36% living at home (*n* = 210) and 64% living apart from parents (*n* = 378).

**Table 1.** Descriptive Statistics for Independent and Dependent Variables (*N* = 588).

Variables	Percentage (*N*)/Mean (SD)
Independent variables	
Child gender	
Female	47 (279)
Male	53 (309)
Child age	28 (4.8)
Child marital status	
Not married/partnered	64 (377)
Married/partnered	36 (210)
Child main activity	
Student	33 (192)
Employed	60 (350)
Other	7 (46)
Child living with parents	
Yes	36 (210)
No	64 (378)
Ethnocultural identity	
British	21 (125)
Chinese	21 (125)
Persian/Iranian	25 (144)
South Asian	33 (194)
Years in Canada	30.3 (18.6)
Parent gender	
Female	57 (336)
Male	43 (252)
Parent age	59.6 (6.6)
Parent marital status	
Not married/partnered	23 (134)
Married/partnered	77 (454)
Parent income satisfaction	3.3 (1.2)
Parent health status	
Poor	5 (28)
Fair	24 (143)
Good	50 (295)
Excellent	21 (121)
Parent religiosity	
At least weekly	31 (180)
Monthly/several times a year	22 (126)
Rarely/not at all	47 (277)
Dependent variables	
Positive parental–child quality	3.9 (.88)
Negative parental–child quality	1.8 (.66)

#### *Dependent Variables*

Two dependent variable scales were used to examine the positive and negative cognitive/emotional appraisals of the parent–child relationship. These scales were created based on the work of Pitzer et al. (2011) who created the Parent Adult Relationship Quality (PARQ) scale. The PARQ consists of two subscales that capture positive and negative aspects of support and interactions between adults and their parents, and regardless of distance factors. It also shows high internal consistency and adequate convergent validity with extant self-report measures and observed behaviors. Use of the PARQ is also argued to improve upon other measures (e.g., solidarity) since it includes negative items, in addition to items assessing conflict. Moreover, Pitzer et al. (2011) argue that the PARQ is a stable and balanced measure of relationship quality over time.

The PARQ scales include four positive and four negative quality items regarding the parent–child relationship and the items are measured on a 5-point Likert scale, where 1 = never, 2 = rarely, 3 = sometimes, 4 = often, and 5 = always. In our study, respondents were asked the following questions: “I will read you some statements to you regarding your ‘study child.” Please indicate, on a scale of 1–5, how often your study child…. acts warm or affectionate toward me; makes demands for favors or other little things from me; is supportive of the decisions I make; acts insensitively or unsympathetically toward me; does favors or other little things for me; acts angry or hostile toward me; questions or doubts my decisions; acts thoughtful or considerate toward me.” Both of these scales demonstrated good reliability in our study (Positive scale: Cronbach's alpha = .80; Negative scale: Cronbach's alpha = .61).

#### Independent Variables

Thirteen independent variables measuring parent–child relationship quality are shown in Table 1. In this table we provide each variable's frequencies, percentages, and other descriptive statistics. These variables are: 1) *child's gender* (female/male); 2) *child's age*; 3) *child's marital status* (not married/partnered; married/partnered); 4) *child's main activity* (student/employed/other); 5) *living arrangement* categorized as living with parents (yes) or not living with parents (no); 6) *ethnocultural identity* (British, Chinese, Persian/Iranian, and South Asian); 7) *years living in Canada* (in years); 8) *parent gender* (female, male); 9) *parent's age* (in years); 10) parent's marital status (not married/partnered; married/partnered); 11) *parent's income satisfaction* (coded on a 5-point scale from 1 ‘not at all satisfied’ to 5 ‘extremely satisfied’ based on responses to the question, “On a scale of 1 to 5, with 1 being, how satisfied are you with your financial situation at the present time?”; to your (study) child?”; 12) *parent's health compared to peers* (poor, fair, good, very good, excellent) using the question: *“*Compared to people your own age, how would you rate your overall physical health at the present time?”; 13) *parent religiosity* (at least weekly/monthly/several times per year/rarely/not at all). A preliminary tabular analysis of the dependent variables and ethnic groups, including gender breakdowns (not shown in Tables; also see prior descriptive summary) showed that the South Asian group exhibited the most distinct associations. Thus, the South Asian group was used as the reference category for comparative purposes in OLS analyses.

### Analytic Strategy

Ordinary Linear Regression (OLR) techniques were used to examine the relationships between the two dependent variables: positive and negative cognitive/emotional appraisals of the parent–child relationship and the 13 key independent predictors. Variables measured on Likert scales (parent health, parent income satisfaction) were utilized as both categorical and continuous predictors. Since the pattern of findings was replicated and the distribution of these variables was close to normal (skewness less than 2.0), these variables were treated in this study as continuous predictors to maximize parsimony. In addition, we included multiplicative interaction effects between parent gender and ethnicity for each dependent variable analysis to explore gendered patterns in intergenerational relationship quality across different ethnocultural groups.

## Findings

Table 2 reports the standardized regression coefficients (betas) for positive and negative relationship quality and the 13 child and parent predictors. The independent variables were entered into a single main effects model and an interaction model. As shown in Table 2, both the main effects and interaction models for positive and negative relationship quality were statistically significant (Model 1 R^2 ^= .16, *p* < .001; and Model 2 R^2 ^= .10, *p* < .001, respectively).

**Table 2.** OLS Regression of Positive and Negative Relationship Quality on Child and Parent Characteristics, and Interactions.

Independent variables	Positive quality *Std β*	Negative quality *Std β*
Child characteristics		
Child gender (ref = male)	.12**	−.02
Child age	−.04	−.07
Child marital status (ref = married/partnered)	−.03	.05
Child main activity (ref = other)		
Student	−.04	−.04
Employed	.02	−.01
Child living with parents (ref = no)	.02	−.05
Parent characteristics		
Ethnocultural identity (ref = British)		
Chinese	−.04	−.06
Persian/Iranian	−.11	.04
South Asian	.17*	−.17*
Years in Canada	−.04	−.10
Parent gender (ref = male)	.16***	.05
Parent age	−.05	.01
Parent marital status (ref = married/partnered)	−.03	−.11*
Parent income satisfaction	.17***	−.10*
Parent health status	.03	−.09*
Parent religiosity	−.02	.05
Ethnicity × Gender (ref = British/male)		
Chinese/female	−.07	.11
Persian/Iranian/female	.21**	.15
South Asian/female	−.04	.16
Model R^2^	.16***	.10***

*Note*. Higher scores represent better parent's health, higher income satisfaction and religiosity.

* p < .05, ** p < .01, *** p < .001.

The first model provides the OLS regression of positive relationship quality and supported main effect associations for three of the thirteen independent variables. Participants with a female child, who are mothers, and those with higher income satisfaction group reported more positive relationship quality scores (β = .12, *p* < .01; β = .16, *p* < .001; and β = .17, *p* < .001, respectively). The remaining variables in this model were not statistically significant. Turning to interactions between ethnicity and gender, Persian/Iranian mothers exhibited a more positive relationship quality with their child compared to the reference group of British fathers (β = .21, *p* < .01).

### Regression of Negative Relationship Quality on Independent Variables

The standardized regression coefficients (betas) for negative relationship quality are also presented in Table 2. The main effects model was statistically significant as previously mentioned, with four statistically significant associations. Participants who identify as South Asian (compared to the British reference group) are less likely to report negative relationship quality (β = −.17, *p* < .05 Also, respondents who are unpartnered are less likely to report negative relationship quality (β = −.11, *p* < .05). In addition, those with higher income satisfaction and better health are less likely to report negative relationship quality (β = −.10, *p* < .001; β = −.09, *p* < .05). Child's gender, age, marital status, main activity, and whether they live with parents were not statistically significant, as well as several parental characteristics (years living in Canada, gender, age, and religiosity). An interaction effect between ethnic identification and gender was not found to be a statistically significant predictor of negative relationship quality.

## Discussion

From a socio-cultural life course approach, this study explores parent–child relationship quality from the perspective of midlife to older-aged parents, focusing on four Canadian ethnocultural groups (British, Chinese, Persian/Iranian, and South Asian) and various child and parental characteristics. A contribution of this study is that it helps to provide a better understanding of the factors that predict positive or negative interactions and sources of support/conflict among some under-studied, culturally diverse families. Our findings add to broader research showing that the quality of family relationships, including social support (e.g., providing love, advice, and care) and strain (e.g., being critical, making too many demands) influences well-being and through different pathways (Thomas et al., 2017).

Focusing on cultural factors (i.e., ethnic identity, years in Canada, and religiosity), some partial support was found for our expectation that socio-cultural variables predict intergenerational relationship quality. Parents who self-identified as South Asian reported having more positive/less negative relationship quality relative to the British-Canadian reference group. With regard to other cultural factors (religiosity and years in Canada), we did not find these factors as significant predictors of parent–child relationship quality.

Although three of the cultural backgrounds under study are commonly conceptualized as collectivist or family-centered (Chinese, Persian/Iranian and South Asian), South Asian older-aged parents reported more positive parent–child relationship quality compared to these groups. These findings are consistent with studies showing that Canadian South Asian parents generally report highly positive family relationships (Mitchell & Dhillon, 2023). Other international studies (e.g., Chadda & Deb, 2013; Jibeen, 2019; Shariff, 2009) also demonstrate that South Asians may generally be less prone than other ethnic groups to report relationship problems. This observation makes it difficult to pinpoint whether our finding reflects highly close-knit families or cultural differences in reporting. For example, prior research on Chinese Canadian families has found that there is a tendency for parents to express more pragmatic and less emotional or neutral feelings about their relationships with their children (e.g., Yue, 2005). Yet, it is plausible that since South Asian families are a firmly entrenched and well-established group in Vancouver with strong cultural familistic traditions, supportive networks, and neighborhoods, many of the issues faced by other recent immigrant groups (i.e., Iranian/Persian) have dissipated over time.

Moreover, some interesting interaction effects were observed. Persian/Iranian mothers are more likely to report positive relationships with their adult children than British fathers. Although this finding is difficult to interpret, prior research (e.g., see Behjati-Sabet & Chambers, 2006) argues that there is a strong cultural norm for Persian/Iranian women to be at the center of family caring as “good mothers” and to intervene in any family conflicts (e.g., especially with fathers). Conversely, research establishes that historically, many fathers from individualistic cultures (e.g., British-Canadians) were raised and socialized into a culture that prioritized instrumental or ‘breadwinner’ fatherhood roles rather than affective, nurturing roles (e.g., see Battams, 2023).

Our analysis also revealed that parents reporting poorer health tend to report more negative relationships with their adult children, although we do not find this association for the positive relationship quality scale. This finding partially supports our hypothesis and lends support to the studies that show that older parents in poorer health might require more support and caregiving from their adult children, leading to more demands, dependency and a reversal of roles (Connidis & Barnett, 2018; Fingerman et al., 2020; Riley & Bowen, 2005). Thus, this role change can cause stress/strain as aging parents grapple with a loss of control and adult children take on more caregiving responsibilities while also juggling their own family and personal lives (Conway, 2019; Morgan & Brazda, 2013).

The findings also support our expectation that parental satisfaction with their income will affect relationship quality since it is known to commonly affect family relationships and well-being (Mitchell et al., 2019). Notably, lower-income satisfaction is typically indicative of limited or constrained economic resources and financial stress and strain, including an inability to adequately provide for one's needs and/or to provide financial support to adult children (Lee et al., 2021). In 2022, Metro Vancouver's living wage is $24.08 per hour for two working parents supporting a family of four (Ivanova et al., 2022) and many families struggle to rent or buy housing, as well as to generally ‘make ends meet.’ Recent immigrant families and others at risk of marginalization may still face even steeper challenges (Ivanova et al., 2022). Therefore, the income satisfaction of aging parents, particularly in the face of the very high cost of housing and living in Metro Vancouver, in tandem with the escalating costs associated with aging families, significantly influences their relationship with their adult child.

Another important insight that emerged from our study is that mothers (vs. fathers) generally report having more positive relationship appraisals, in addition to parents with daughters rather than their sons, although this association was not found for the negative relationship scale. These findings partially support our hypothesis and previous research that mother–child and parent–daughter relationships tend to be more emotionally close (e.g., see Alford, 2021; Cao et al., 2019; Golish, 2000; Rastogi & Wampler, 1999; Shapiro, 2004). It also highlights the pervasiveness and reproduction of gender role socialization in terms of family norms and behaviors. Moreover, given that our sample is mainly comprised of parents from more traditionally ethnic-based cultures, there may be more pressure for their daughters to conform to strict gender roles, familial divisions of labor, and patriarchal power, such as obedience, thereby mitigating the effects of intergenerational conflict (e.g., see Cheng & Wilmoth, 2023; Cox & Ephross, 1998). Conversely, sons may be given more freedom and latitude, and this may affect factors related to communication patterns, contact, emotional closeness and affection, support, and intergenerational conflict.

Finally, another important finding gleaned from our analysis is that unpartnered parents (e.g., divorced, widowed) tend to report more positive relationships with their children than partnered parents (i.e., married). Yet, this association was not found for the negative relationship scale. This observation lends partial support to our hypothesis and idea that changes in marital roles and status for aging parents can have positive effects. For example, divorced or widowed parents may lean more on their adult children for companionship and emotional support, potentially deepening the relationships.

In summary, our analysis reveals that from the parental perspective, relationship quality with children is diverse and is shaped by ethnic background, parental health status, satisfaction with finances, gender of the parent and the child, and parental marital status. Interaction effects were also found between ethnic identification and gender. Contrary to our initial hypotheses, we did not find associations for the other variables with the two scales included in the analysis (child's age, child's marital status, child's main activity, living with parents, parent's years in Canada, parent's age, parent's religiosity). Overall, these findings demonstrate the value of applying a socio-cultural life course perspective to better understand diversity in mid/later-life Canadian ethnic families and how ethnocultural, gendered, and personal lives are embedded in relative time and place, as well as aging-related developmental circumstances.

## Conclusion

This study offers a unique exploration of parent–child relationship quality among older-aged parents from different ethnic groups living in Vancouver, Canada, including British, Chinese, Persian/Iranian, and South Asians. The findings shed light on the complex interplay of socio-cultural factors, individual characteristics, and relational dynamics that contribute to these intergenerational relationships.

### Limitations and Future Research

One limitation of this study is generalizability since we focused on a specific set of ethnic groups within a particular geographic context (British Columbia, Canada). Thus, these findings might not resonate with these ethnic groups living in other parts of the world as well as other diverse ethnic groups living within Canada. Also, our inclusion criteria included only those who primarily identify with one of the four ethnic groups under study. Yet, ‘Canadians’ are increasingly likely to identify with a wide array of racialized or multiple ethnic groups (Statistics Canada, 2022), which can potentially shape parent–child interactions and relationships in unique ways.

Additionally, the method of data collection raises concern for self-report bias. Social desirability is a significant concern in family research, as it can potentially bias the results and distort the true nature of family dynamics and behaviors. The reliance on self-reported data might introduce bias due to social desirability and memory recall (Bell & Bell, 2018; Johnson & Van de Vijver, 2003; Krumpal, 2013). Cultural norms and expectations could influence how participants report their relationships and how they want them to be perceived. Due to distinct sets of values, beliefs, and behavioral expectations to adhere to these norms, participants may perceive and report experiences that align with what they perceive as ‘socially acceptable’ within their cultural context (Johnson & Van de Vijver, 2003; Lalwani et al., 2006; Saris & Gallhofer, 2007). For instance, Lalwani et al. (2006) suggest that people from both individualist and collectivist societies exhibit socially desirable responses, albeit through different approaches; individualism tends to exaggerate skills, whereas collectivism emphasizes positive self-presentation.

Relatedly, the sample of each ethnic group might not fully represent the diversity ‘within diversity.’ Characteristics such as socio-economic status and gender can affect meanings and understandings of social and physical well-being in relation to parent–child dyads. Thus, there may be diversity within the ethnic groups under study (e.g., among South Asians, due to differing immigration histories, and socio-economic statuses) that we were unable to capture. For example, our preliminary analysis (not shown here) did not find that parental educational attainment was a significant predictor of relationship quality, although more fine-grained research could reveal sub-group differences within each cultural group. These factors are supported by the effects of the social determinants of health (Raphael, 2016) and an intersectionality approach to family research (Few-Demo, 2014; Few-Demo & Allen, 2020).

Also, our cross-sectional design limits the ability to establish causality or demonstrate changes in relationship quality over time (Wang & Cheng, 2020). Moreover, key developmental changes within the parent–child relationship may have been missed or may not account for the shifting dynamics as family members age and gain new roles (e.g., becoming grandparents), or face challenges and transitions. To address the limitations of this design, future research should adopt longitudinal research strategies to allow for the examination of changes in relationship quality. Follow-up can provide deeper insights into the dynamics of changing parent–child interactions and their shifting contexts.

Other research directions should also include the collection of qualitative data. Qualitative studies illuminate the internal and subjective dynamics of family relationships. For instance, Alford's (2021) study with in-depth interviews with adult daughters illustrates the active role they play in nurturing a thriving mother-daughter relationship. Other qualitative approaches, such as ethnographic fieldwork of LGTBQ spaces, showcase how gay and lesbian adults manage their parents’ presence through “comfort work”, resulting in meaningful acceptance (Stone, 2021). These studies highlight the richness of qualitative research in understanding complex relational dynamics and illuminate social processes that shape the parent–adult child dyad. Combining qualitative methods, such as interviews, observations or focus groups, with quantitative data could offer a richer and more comprehensive understanding of cultural influences and individual experiences.

Furthermore, qualitative explorations can highlight the personal lived experiences and narratives of individuals within these ethnic groups. This approach can uncover distinct cultural practices and subjectivities associated with changes in parent–child relationships, shifts in family dynamics, or the effects of certain life events within the family. Also, large-scale comparative cross-national studies across different countries and cultures should be considered as a future direction. This approach could provide a broader perspective on the impact of societal and policy environments (e.g., related to family caregiving), gendered cultural norms, migration, and socio-economic factors on parent–child relationships.

Expanding the focus of this study to include the perspectives of (all) adult children is another promising direction for future research. Our study was limited to the inclusion of data (as reported by parents) on one child only, and their generational perspective was not included in this study. The inclusion of data collected from younger generations would provide a more comprehensive picture of these intergenerational dynamics. Future research also needs to fully investigate a wider range of instrumental and affective intergenerational transfers and generational discrepancies in issues related to reciprocity, equity, and fairness. Relatedly, it is also possible that parents with multiple children may report mixed experiences, both higher and lower quality, contributing to ambivalence in parent–child relations (Ward, 2008; Ward et al., 2009). These variable relationships may have significant implications for support exchanges. Moreover, for partnered parents, it has been found that their relationship quality is associated with the quality of parent–child relationships (e.g., see Mitchell & Dhillon, 2023). Thus, future studies could focus on how parents with marital stress or difficulties (e.g., related to rigid gender roles and a strict division of labor) might affect relationships with children.

Thus, future research should focus on cultural norms and values, in addition to family structure, and how these factors influence social interactions, contact patterns, and support exchanged between older parents and their adult children. Moreover, understanding how transnational caregiving, where adult children may provide caregiving assistance across international borders, impacts aging relationships is essential, given the increasing globalization and migration patterns of families (Miyawaki & Hooyman, 2023). Exploring these areas can illuminate the evolving nature of familial obligations, roles, responsibilities and struggles across generations and geographical boundaries. Additionally, future studies should incorporate how advancements in communication technologies (e.g., video chats, and digital messaging) influence contemporary aging family life and the quality of intergenerational relations.

### Policy and Practical Implications

The findings from this study offer implications for both policy development and practical interventions that can further support and improve parent–child relationship quality among aging families from diverse cultural backgrounds. By considering the nuanced interplay of socio-cultural factors, individual characteristics, and relational dynamics, policymakers and practitioners can tailor their approaches to better address the unique needs of these families to improve their health and well-being.

In terms of applied implications, observations gleaned from this study can be used to further emphasize the need for policymakers to encourage cultural sensitivity and competence training for professionals working with aging families. Current efforts in policy development and resource allocation have addressed critical issues pertaining to rapid population aging, specifically focusing on the provision of support for family caregivers (Family Caregivers of British Columbia, 2023), assisting older adults in navigating the continuing care sector (BC Care Providers Association, 2022), fostering services to encourage aging-in-place initiatives (Government of British Columbia & United Way, 2023) and mitigating elder abuse and neglect (Fraser Health, 2023). These initiatives and resources have significant implications for improving relationship quality between older parents and their adult children.

Despite these advancements, however, there remains a need to more fully acknowledge and accommodate diverse family structures when developing policies and resources that cater to aging families. For instance, this study highlights that unparented parents tend to report more positive relationships. Therefore, policies should consider the needs of divorced, widowed, and unpartnered parents and how support groups, counseling services, and community resources may vary in light of this diversity in family structure. Also, given that older parents with better health tend to report more positive relationships, policies and programs that provide navigational support to healthcare services and preventive care for aging populations are crucial.

It is also important to more clearly recognize the negative impact of income dissatisfaction on relationship quality. Policies could focus on improving financial support (e.g., tax credits) for both older-aged parents and their adult children who might be taking on more caregiving responsibilities. They could also offer additional individualized and family-based needs assessments, provide expanded respite services, and remove financial barriers for low-income individuals and families (e.g., see National Seniors Strategy, 2020).

In terms of practical interventions, initiatives that promote healthy intergenerational interactions, such as workshops, storytelling sessions, or joint activities, can facilitate understanding and strengthen bonds between older parents and their adult children. Also, interventions that integrate cultural values and practices into their design could resonate more effectively with families from different ethnic backgrounds. These programs could focus on enhancing communication, conflict resolution, and emotional expression in relation to cultural norms and values. One example of this type of intervention is the “Relationships with your Adult Children Group” in Calgary, AB which includes insights from experienced facilitators to address evolving family dynamics and provide education, support, and resources for older parents to manage their ties with their adult children (Carya, 2023). Other interventions could include providing access to family mediation and counselling services tailored to the cultural context of the family.

Mediation and counselling can help address conflicts and facilitate healthier communication patterns within parent–child relationships. For instance, Family Meditation Canada (2022) offers a range of services including webinars, events, and professional support, including cultural differences (generational, ethnic, gender, etc.) as an approach. Also, workshops or programs targeted at specific moderating factors, such as finances, can be a promising avenue for strengthening this relationship. For instance, supporting older parents in managing their finances and planning for their future can contribute to increased financial satisfaction, thereby reducing potential stressors that impact parent–child relationships.

In conclusion, this study offers a unique and timely exploration of parent–child relationship quality among ethnically diverse older-aged parents. By considering the influence of socio-cultural factors, individual attributes, and relational dynamics, the findings underscore the complexity of these intergenerational relationships. While highlighting limitations and suggesting a direction for future research, this study emphasizes the need to further examine the evolving nature of parent–child interactions within the context of changing immigration and cultural norms, health considerations, financial circumstances, and gender roles. By informing both policy development and practical interventions, this research contributes to the promotion of healthier and more supportive relationships that enhance family well-being, aging, and cultural cohesion.
